# The Size Effects of Point Defect on the Mechanical Properties of Monocrystalline Silicon: A Molecular Dynamics Study

**DOI:** 10.3390/ma14113011

**Published:** 2021-06-02

**Authors:** Wei Wan, Changxin Tang, An Qiu, Yongkang Xiang

**Affiliations:** Institute of Photovoltaics, Nanchang University, Nanchang 330031, China; 5701118206@email.ncu.edu.cn (W.W.); 5701118192@email.ncu.edu.cn (A.Q.); 5904118078@email.ncu.edu.cn (Y.X.)

**Keywords:** monocrystalline silicon, molecular dynamics, point defect, mechanical properties

## Abstract

The molecular dynamics method was used to simulate the fracture process of monocrystalline silicon with different sizes of point defect under a constant strain rate. The mechanism of the defect size on the mechanical properties of monocrystalline silicon was also investigated. The results suggested that the point defect significantly reduces the yield strength of monocrystalline silicon. The relationships between the yield strength variation and the size of point defect fitted an exponential function. By statistically analyzing the internal stress in monocrystalline silicon, it was found that the stress concentration induced by the point defect led to the decrease in the yield strength. A comparison between the theoretical strength given by the four theories of strength and actual strength proved that the Mises theory was the best theory of strength to describe the yield strength of monocrystalline silicon. The dynamic evolution process of Mises stress and dislocation showed that the fracture was caused by the concentration effect of Mises stress and dislocation slip. Finally, the fractured microstructures were similar to a kind of two-dimensional grid which distributed along the cleavage planes while visualizing the specimens. The results of this article provide a reference for evaluating the size effects of point defects on the mechanical properties of monocrystalline silicon.

## 1. Introduction

Monocrystalline silicon exhibits excellent photoelectric performance, doping characteristics and chemical properties due to its microstructure, and is commonly used in the manufacturing of large-scale integration and photovoltaics. The monocrystalline silicon was cut from silicon ingots by wire saw [1] as the raw materials of these products. However, in the production of silicon ingot, there is edge collapse, hidden crack, debris and other defective products [2], which reduce the utilization rate of raw silicon, increase the economic cost and hinder the development of the monocrystalline silicon industry. Therefore, there is a need to investigate and improve the mechanical properties of the monocrystalline silicon product.

Many scholars have carried out research on the mechanical properties of silicon. Liu et al. [3] used the indentation method to investigate the deformation and surface damage of monocrystalline silicon, combined with the results given by molecular dynamics simulation to verify the deformation theories of crystal substructure. Because molecular dynamics simulation provides detailed information about the evolution of atomic microstructures, it is also a powerful research tool for understanding the properties of materials. During the decades of development of the monocrystalline silicon industry, through theoretical analysis, experimental tests and computational simulations, the reinforcement effect of nitrogen [4], oxygen [5] and dislocation [6] on the mechanical properties of monocrystalline silicon and the mechanisms of these impurities above have been fully comprehended by the research community. However, due to negative factors, such as temperature, thermal stress and rapid crystallization, point defects are formed in the production of monocrystalline silicon. Thus, the point defect should be considered in order to completely discuss the factors which may affect the mechanical properties of monocrystalline silicon. At present, many outstanding works have been carried out through theory, experiments and simulation methods in the research of point defects on the mechanical properties of metals [7,8], composites [9,10,11] and carbon-based materials [12,13]. The effects of crystal defect were pointed out by Wu et al. [14] on the mechanical properties of multicrystalline silicon, which is the main competitor of monocrystalline silicon. The silicon defects induced by some experimental methods, such as spot laser melting [15] and directional solidification [16], were also carried out by some researchers. As for the mechanical properties of monocrystalline silicon, the size effects of point defects at the nanoscale view of molecular dynamics have not been reported yet.

On the other hand, the monocrystalline product of silicon gained wide application in new electrode materials, such as silicon anode, for its high theoretical gravimetric capacity and being environmentally friendly. However, volume expansion will appear in the lithiation/delithiation process if the anode is based on silicon. This results in low coulomb efficiency and capacity fading [17]. Thus, the suppression of volume expansion in the lithiation/delithiation process has become very crucial for current silicon anode research. To solve this problem, Darbaniyan et al. [18] investigated the effects of strain rate and mass fraction of lithium on the mechanical properties of silicon crystals. The results revealed two potential factors affecting the mechanical properties of silicon anode. Kim et al. [19] successfully suppressed the volume expansion by pre-lithiation. Some researchers focused on the structure design of anode, proposing multiple optimization methods to improve the mechanical properties of silicon anode. For example, Han et al. [20] showed a mechanical buffer enhancement strategy to stabilize the carbon/silicon surface through controllable shrinkage combined with a carbon cage network in the construction of the silicon anode. The volume expansion of lithiation was effectively eliminated. Xie et al. [21] encapsulated the micro/nano-sized silicon particles into a nitrogen-enriched porous carbon matrix, using CaCO_3_ as the structural template and polyacrylonitrile (PAN) as the carbon and nitrogen source. The resultant honeycomb shaped porous composites exhibited a dramatically enhanced cycling stability and excellent rate performance, which could well adapt the volume expansion of the lithiation/delithiation process. However, researchers have not discussed the variation of mechanical properties in monocrystalline silicon under the effects of defect cluster by the molecular dynamics method from the micro perspective.

In the present paper, a molecular dynamics method, combined with a typical potential function of silicon, was used to simulate the tensile fracture process of monocrystalline silicon under a constant strain rate. By generating point defects with different atomic sizes in the crystals, the size effects of point defects on the mechanical properties of monocrystalline silicon were discovered. Further discussions and analysis suggested that the stress concentration caused by point defects decreased the yield strength, which is the mechanism of strength reduction. The Mises theory was verified by stress computation, which proved to be the best theory of strength to describe the mechanical properties of monocrystalline silicon. The dislocation analysis suggested that the dynamic dislocation behaviors also existed in the crack extension process during the crystal fracture. It proved that the dislocation behaviors are also a major reason for the fracture. In the yield stage of monocrystalline silicon, the microstructures formed by the fractured planes were similar to a kind of two-dimensional grid. This phenomenon indicated that the microstructures and conditions of fracture had certain regularity. Finally, in the monocrystalline silicon production of the photovoltaic industry, the existence of defects with different sizes form in crystals [22] and decrease the yield strength, which may cause edge collapse, hidden cracks and debris while cutting silicon wafers. Such strength decays are more serious for defects with larger sizes. Therefore, the authors expected to provide a reference for further research studies about crystal defects on the mechanical properties of monocrystalline silicon and the quality improvement of the silicon wafer cutting process.

## 2. Model and Methods

**Figure 1:**
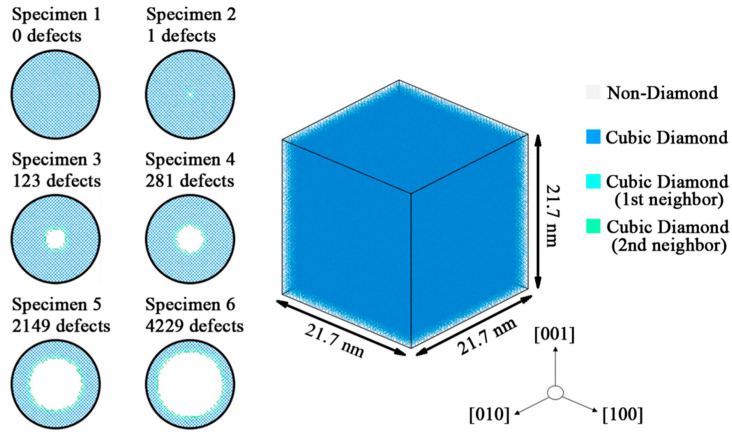
Cell model of monocrystalline silicon and 6 specimens with point defects.

The lattice constant of silicon is 0.543 nm. An ideal, diamond structure monocrystalline silicon crystal was generated by LAMMPS (Large-scale Atomic/Molecular Massive Parallel Simulator). The simulation box had a size of 21.7 × 21.7 × 21.7 nm^3^ along the X, Y, and Z directions, respectively, containing about 512,000 silicon atoms. The X, Y and Z axes respectively corresponded to the [100], [010] and [001] crystal directions of the monocrystalline silicon. The periodic conditions were used in all directions of the system boundary during the simulation, for the periodic boundary condition greatly eliminated the surface effects [23]. Figure 1 shows the ideal crystal and 6 specimens with point defects. Spherical regions with different radius were divided in the geometric center of the ideal crystals. The atoms in these regions were deleted to generate the point defects. The size of the point defect was measured by the amount of deleted silicon atoms. Table 1 shows detailed information about the size of the point defect in each specimen.

Then, the crystals were thermally equilibrated to 300 K for 300 picoseconds, using isobaric/isothermal constant number of particles, constant pressure and constant temperature (NPT) ensemble, so the total stress became zero. The timestep was set to 1 femtosecond. During dynamic loading, the crystals were subjected to uniaxial tensile load with a constant strain rate, 1 × 10^8^/s^−1^, along the X direction, whereas the pressure in both Y and Z directions was kept at zero by using a Berendsen [24] barostat. The temperature was controlled every timestep by using a Berendsen thermostat. The Verlet algorithm was used to calculate the trajectory of the atoms. Visualization of all molecular dynamics simulation snapshots were made via the open source software Ovito (2.6.1) [25].

The interaction of silicon atoms is described by Tersoff potentials. The parameters of the potential were developed by fitting to the experiment data of the silicon systems. The Tersoff potential is a kind of bond potential, first reported in 1986, revised twice in 1988 and 1989. It had three versions: T1 [26], T2 [27] and T3 [28]. The T2 version of Tersoff potential (Tersoff.mod) was used in this simulation, for it describes not only the structure of diamond, but also the non-tetrahedral structures of silicon, such as cluster, crystal orientation and liquid silicon. The defect formation energy of silicon is 7.3 eV under the Tersoff potential (T2), which shows a superiority in describing the properties of diamond silicon, compared with other potential functions [29]. For example, Zhou et al. [30] simulated the melting characteristics of silicon crystal under the effects of Tersoff, SW and MEAM potentials. The results suggest that the Tersoff potential is better for describing the melting process of silicon.

## 3. Results

### 3.1. The Size Effects of Point Defect on Mechanical Performance

**Figure 2:**
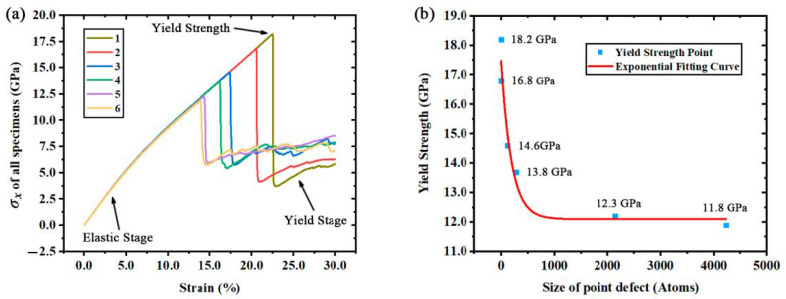
(**a**) Stress-strain curves of all specimens. (**b**) Exponential fitting curve of yield strength and defect size.

Crystal easily forms defects, which critically reduce its mechanical properties [31], due to the thermal stress and other factors in its formation. To analyze the size effects of point defects on the mechanical properties of monocrystalline silicon, the stress–strain curves of all specimens during the deformation are plotted in Figure 2a. The stress–strain curve of ideal crystal shows the tensile process of monocrystalline silicon, including the elastic stage and yield stage. In the elastic stage, the stress level ascends linearly as the strain increases; the deformation in this stage is elastic and restorable. All specimens show the same deformation behaviors in the elastic stage, which may be due to the potential. The fracture appears when the stress exceeds the yield strength. Then, the stress decreases rapidly to the lower yield point and turns to the yield stage. Comparing different stress–strain curves, it is found that both the yield strength and the maximum strain of monocrystalline silicon are significantly reduced by point defects.

As shown in Figure 2b, while doing some statistical work about the effects of point defects on mechanical properties, it is found that the size of point defects and the yield strength $\sigma_{s}$ follow an exponential function:

(1) $$\sigma_{s}(c)=\sigma_{0}+A\times Exp(R\times c)$$

where *c* is the size of point defect, *σ*_0_ is the minimum yield strength, which is approximately equal to 12.0504 ± 0.4568 GPa, *A* = 5.4136 ± 0.6398 GPa, *R* = −0.0051 ± 0.0015. The correlation coefficient of this exponential fitting is equal to 0.9998 and the residual sum of squares is equal to 0.4253. Among all kinds of exponential fitting functions, the presented function is the best and clearest with the lowest error range of parameter *R*. Both *A* and *R* are parameters related to the defect properties. Further research is required for the investigation of factors which may affect *A* and *R* parameters.

### 3.2. Discussing the Mechanism of Strength Reduction from Stress Variation

**Figure 3:**
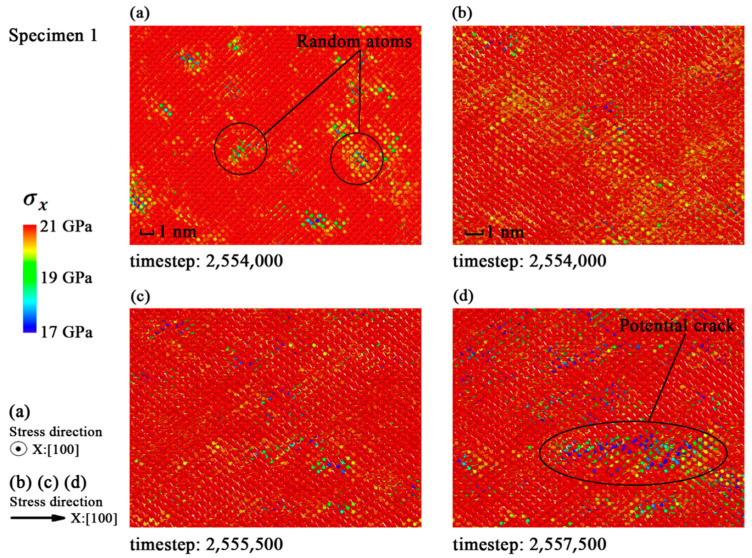
Distribution of *σ*_x_ in specimen 1 before fracture: (**a**) Snapshot at timestep 2,554,000; (**b**) snapshot at timestep 2,554,000; (**c**) snapshot at timestep 2,555,500; (**d**) snapshot at timestep 2,557,500.

**Figure 4:**
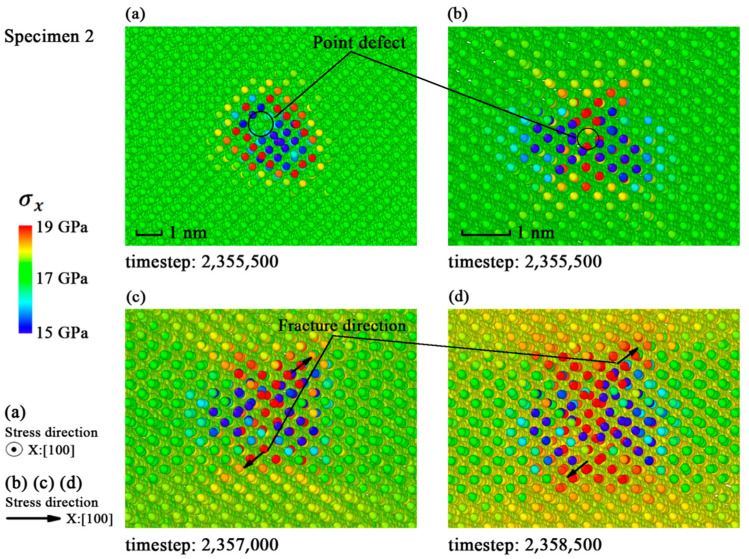
Distribution of *σ*_x_ in specimen 2 before fracture: (**a**) Snapshot at timestep 2,355,500; (**b**) snapshot at timestep 2,355,500; (**c**) snapshot at timestep 2,357,000; (**d**) snapshot at timestep 2,358,500.

**Figure 5:**
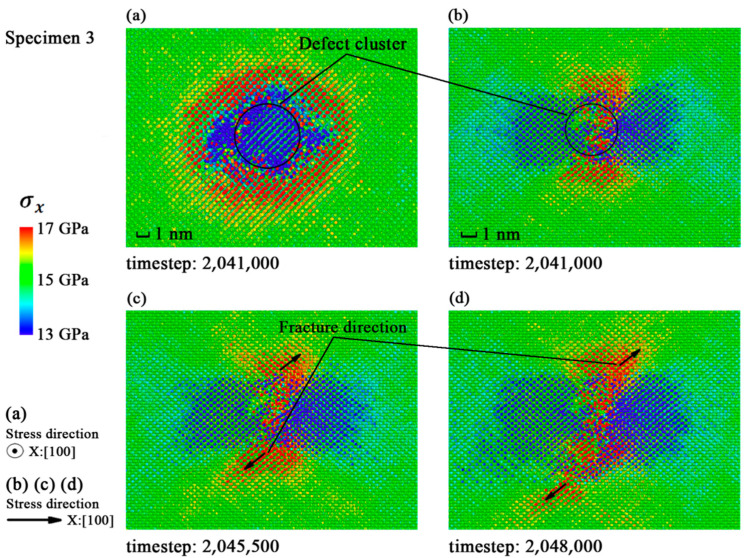
Distribution of *σ*_x_ in specimen 3 before fracture: (**a**) Snapshot at timestep 2,041,000; (**b**) snapshot at timestep 2,041,000; (**c**) snapshot at timestep 2,045,500; (**d**) snapshot at timestep 2,048,000.

Bullegas et al. [32] studied the effects of stress concentration on the fracture process of composites and concluded that the concentration and the release of internal stress were the main causes of fracture. To discuss the fracture mechanism of monocrystalline silicon, the stress tensors at a period of 500 timesteps of each atom were used to plot the distribution of stress in X direction (*σ*_x_) as shown in Figure 3, Figure 4 and Figure 5.

The *σ*_x_ in specimen 1 shows a random distribution in Figure 3a, while the cracks may appear in random places due to the uniformity of ideal crystal. Figure 4a and Figure 5a indicate that the distributions of *σ*_x_ in the specimens with single defect and larger defect clusters are roughly the same. The stress is concentrated on dangerous sections perpendicular to the strain loading direction. The snapshots in Figure 4b–d and Figure 5b–d show that the fracture directions of different specimens are all along the (111) crystal plane. So, the <111> crystal family is the major concern in the next subsection.

**Figure 6:**
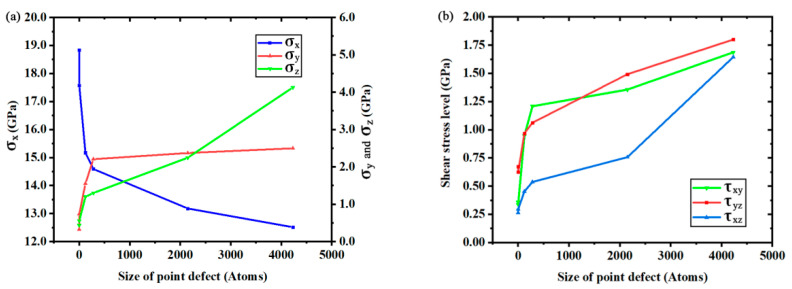
The relationship between maximum stress level and the size of the point defect within 1 picosecond before fracture: (**a**) tensile stress; (**b**) shear stress.

Figure 6 quantitatively shows the relationship between the maximum stress level and the size of point defect. The variation of *σ*_x_ suggests that the maximum tensile stress inside the crystal is always greater than the yield strength shown in Figure 2b. They both have the same trend of variation. Despite the upward trend of tensile stress in the Y direction (*σ*_y_) and tensile stress in the Z direction (*σ*_z_), the curve of *σ*_x_ in Figure 6a suggests that the fracture may depend on the value of *σ*_x_. However, the increasing trend of other stress tensors (shear stress in the XY plane: *τ*_xy_; shear stress in the YZ plane: *τ*_yz_; shear stress in the XZ plane: *τ*_xz_) in Figure 6a,b shows that the combined result of all stress tensors should be considered as the reason of fracture. To verify the existence of such a complex stress condition above in the fracture process of silicon crystal under uniaxial tension, the equivalent stress on each atom was calculated by using atomic stress tensors [33] according to the formulas [34] of four theories of strength.

**Figure 7:**
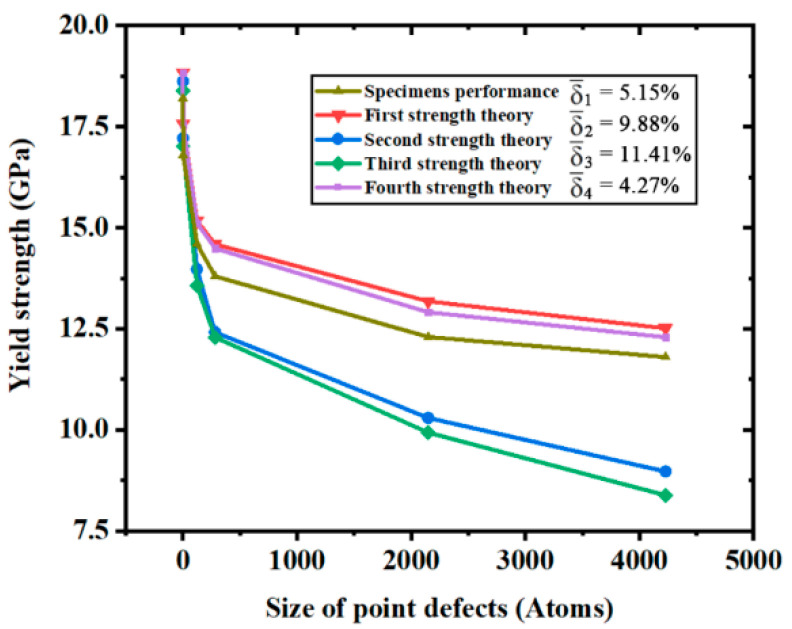
Theoretical strength given by four theories of strength compared with actual specimen performance.

Researchers have already developed many theories of strength to reveal the fracture mechanics of multiple material types [35,36] according to their engineering requirements [37]. Amongst all the theories, the most famous theories are four theories of strength mentioned by the textbooks about engineering mechanics. Figure 7 gives the yield strength of specimens and theoretical strength of the four theories. Average relative error ($\bar{\delta }$) and theoretical strength under larger defect sizes show that the second theory of strength (in this theory, the fracture is caused by maximum tensile strain) and the third theory of strength (in this theory, the fracture is caused by maximum shear stress) do not match the variation of yield strength precisely. The theoretical strength given by the first theory of strength (in this theory, the fracture is caused by maximum tensile stress) and the fourth theory of strength (Mises theory) are well consistent with the actual strength variation. However, the first theory of strength does not take the variation of other stress tensors, shown in Figure 6, into consideration. So, the fourth theory of strength was regarded as the best theory of strength to describe the yield strength of monocrystalline silicon. Considering the combination effects of tensile stress and shear stress, the fourth theory of strength was proposed by Von Mises based on elastic strain energy. It is also a distortion energy density theory, which is used to calculate the yield strength of materials. The equivalent stress according to the Mises theory is called Mises stress. The formula of yield strength in the Mises theory is as follows:

(2) $$\sigma_{s}=\sqrt{\frac{1}{2}({\left(\sigma_{x}-\sigma_{y}\right)}^{2}+{\left(\sigma_{y}-\sigma_{z}\right)}^{2}+{\left(\sigma_{z}-\sigma_{x}\right)}^{2}-6(\tau_{xy}^{2}+\tau_{yz}^{2}+\tau_{xz}^{2}))}$$

where *σ*_s_ is the Mises stress given by the stress tensors of an atom. When the Mises stress exceed a critical value, the atoms will start to the plastic state or fracture.

**Figure 8:**
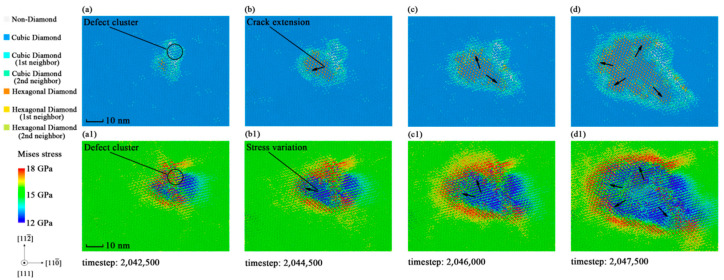
Crack variation and Mises stress variation of crystal plane in specimen 3 during the process of fracture: (**a**) Crack snapshot at timestep 2,042,500; (**b**) crack snapshot at timestep 2,044,500; (**c**) crack snapshot at timestep 2,046,000; (**d**) crack snapshot at timestep 2,047,500; (**a1**) Mises stress snapshot at timestep 2,042,500; (**b1**) Mises stress snapshot at timestep 2,044,500; (**c1**) Mises stress snapshot at timestep 2,046,000; (**d1**) Mises stress snapshot at timestep 2,047,500.

The fracture directions showed in Figure 4 and Figure 5 are along the <111> cleavage plane family. The fracture phenomenon of this plane family is more representative for revealing the reasons of fracture. Figure 8 shows the distribution and variation of Mises stress on $\left(1\bar{1}1\right)$ crystal plane according to Equation (2) with the images of crack extension. Black arrows are used to indicate the directions of crack extension and Mises stress variation. Different from the distribution of *σ*_x_, Mises stress shows an obvious non-uniform distribution, which gives a better explanation for the formation and the extension of cracks. Figure 8(b1,c1,d1) suggests that the variation of the fracture regions are always accompanied by the regional extension of Mises stress concentration. Fractures will not appear in regions where the Mises stress level does not reach the Mises theoretical strength. It results in the directed crack extension in Figure 8.

After the recognition of the phenomenon that the point defect caused on the mechanical performance of monocrystalline silicon, the mechanism of strength reduction is now concluded as follows: the point defect will change the stress distribution in the crystal by concentrating the stress in its surrounding. The fracture condition will conform to the Mises theory under the effects of the point defect. Therefore, the variation of the actual yield strength matches the predicted trend of the Mises theory. This mechanism suggests that the shear stress also participates in the fracture, which also provides a theoretical reference for evaluating the mechanical properties of monocrystalline silicon.

### 3.3. Dynamic Evolutions of Dislocations in Fracture Process

Yonenaga et al. [38,39] investigated the dislocation dynamics in the deformation of silicon crystals via theoretical analysis and experiments. They concluded that the stress–strain characteristics during yielding are controlled by the dislocation processes occurring during such a transient period. Until now, computer modeling and the experimental analysis [40] of silicon dislocations have proved such points as those mentioned above. Thus, from the dislocation dynamics view, the dynamic evolutions of dislocations in the fracture process of silicon were completed by using the dislocation analysis (DXA) function of Ovito.

**Figure 9:**
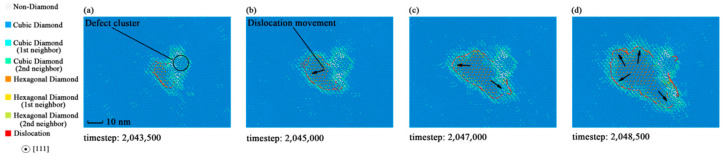
Dynamic behaviors of dislocation in specimen 3 during the process of fracture: (**a**) Dislocation snapshot at timestep 2,043,500; (**b**) dislocation snapshot at timestep 2,045,000; (**c**) dislocation snapshot at timestep 2,047,000; (**d**) dislocation snapshot at timestep 2,048,500.

In the elastic stages, there was no dislocation existing in the specimens, but as the crack appeared, dislocations formed too. Figure 9 shows the dislocation evolution in the fracture process of monocrystalline silicon. The dislocation was distributed along the boundaries of the fractured regions. These results indicate that the crystal crack will form dislocations while under tensile stress loading. As the cracks are extended, the dynamic behaviors of dislocations perform the same trend with the crack boundaries. This provides another explanation for the fracture phenomena in monocrystalline silicon.

**Figure 10:**
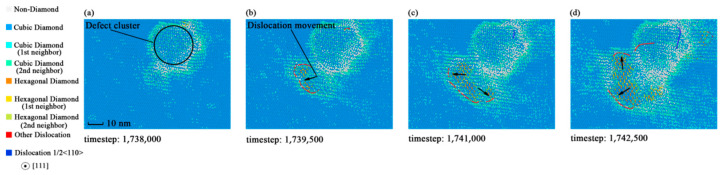
Dynamic behaviors of dislocation in specimen 5 during the process of fracture: (**a**) Dislocation snapshot at timestep 1,738,000; (**b**) dislocation snapshot at timestep 1,739,500; (**c**) dislocation snapshot at timestep 1,741,000; (**d**) dislocation snapshot at timestep 1,742,500.

However, the dislocations of specimen 5 showed in Figure 10 are more discontinuous and shorter, compared with specimen 2. This indicates that the yield strength reduction depends on the dislocation extension or slip. Specimens with larger defect sizes are close to the minimum yield strength of silicon, for they have fewer dislocations. As for the ideal crystal, dislocations may be generated at the slip planes inside the crystal due to the uniformity. The dislocation dynamics analysis of monocrystalline silicon provides a new aspect to reveal the mechanism of yield strength reduction.

### 3.4. Fractured Microstructures of Monocrystalline Silicon

**Figure 11:**
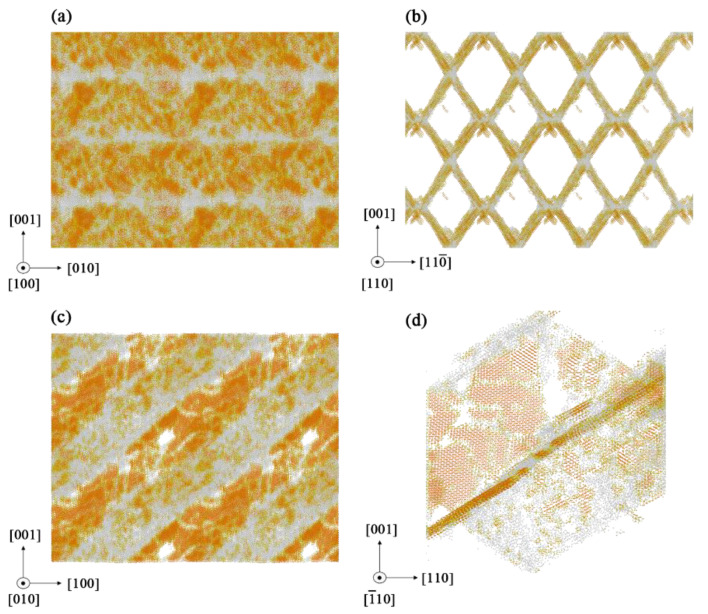
Fractured microstructures caused by tensile stress from (100) crystal direction in monocrystalline silicon: (**a**) Camera view of (100) direction; (**b**) camera view of (110) direction; (**c**) camera view of (010) direction; (**d**) grid structure in single cell model.

As mentioned in Section 3.2, the fractured microstructures of the specimens in Figure 11 show a certain regularity in <111> crystal plane family by removing the silicon atoms. Figure 11a,c shows periodic structural repetition at (100) and (010) planes. The fractured structures in Figure 11b are similar to a non-orthogonal and two-dimensional grid. The hexagonal diamond atoms are mainly distributed on the $\left(1\bar{1}\bar{1}\right)$ and $\left(11\bar{1}\right)$ cleavage planes of the two-dimensional grid, while the amorphous silicon atoms are mainly distributed on the edge of $\left[1\bar{1}0\right]$ direction where the two crystal planes intersect. Figure 11d shows that only two crystal planes appear in a single specimen.

Table 2 shows the distribution of fractured planes in different specimens. There are four possible fracture planes in <111> crystal family, which are (111), $\left(11\bar{1}\right)$, $\left(1\bar{1}1\right)$ and $\left(1\bar{1}\bar{1}\right)$ planes. The angle between these planes and the initial tensile direction is 54.74°. However, only two crystal planes with an angle of 70.53° will appear randomly in each specimen. Because it is found that the stress tensors on two of the four fracture planes with an angle of 70.53° match the fracture condition in formula (2), the fracture will appear on the two planes.

## 4. Conclusions

The size effects and the mechanism of the point defect on the mechanical properties of monocrystalline silicon were investigated by molecular dynamics simulation. The main preliminary results are summarized as follows:

The variation of yield strength shows that the point defect reduced the yield strength of monocrystalline silicon. The relationship between the yield strength and the size of the defect are in accordance with the exponential function shown in Formula (1). The mechanism of the yield strength reduction caused by the point defect is that the point defect will change the stress state of the crystal by inducing stress concentration in its surroundings. By giving the theoretical yield strength of four theories of strength, the Mises theory is proved to be the best theory of strength to calculate the mechanical performance of monocrystalline silicon. The evolution of Mises stress also matches the crack extension in the crystal. Dislocation analysis was used to reveal the dynamic evolution process of silicon dislocations, which proved to be another reason for the crack extension and one of the possible explanations for the yield strength reduction. Then, the microstructures of fracture were visualized by Ovito, and it was found that the fractured silicon structures were similar to a kind of two-dimensional grid along the cleavage planes of silicon. The visualization results indicate there are four possible fracture planes in monocrystalline silicon; once the stress tensors on two planes with an angle of 70.53° reach their limit, a fracture will appear in the two planes.

## Figures and Tables

**Table 1:** The size of point defect in different specimens.

Specimen Identifier	Number of Deleted Atoms	Radius of Point Defect/nm
1	0	0.000
2	1	0.0543
3	123	0.0815
4	281	1.086
5	2149	2.172
6	4229	2.715

**Table 2:** Distribution of crystal planes in the fractured microstructures.

Specimen Identifier	Crystal Plane 1	Crystal Plane 2	Angle between Two Planes
1	(111)	$\left(1\bar{1}1\right)$	70.53°
2	$\left(1\bar{1}\bar{1}\right)$	$\left(11\bar{1}\right)$	70.53°
3	$\left(1\bar{1}\bar{1}\right)$	$\left(1\bar{1}1\right)$	70.53°
4	(111)	$\left(1\bar{1}1\right)$	70.53°
5	(111)	$\left(1\bar{1}1\right)$	70.53°
6	(111)	$\left(1\bar{1}1\right)$	70.53°

## Data Availability

Data available on request due to restrictions like data capacity. The data presented in this study are available on request from the corresponding author. The data are not publicly available due to data capacity (According to an uncompleted statistic, the related simulation data in the present paper is about at least 50 GB) which is too big to storage for the website. Please inform the corresponding author for further usage of the related simulation data.
